# Development of Theophylline Microbeads Using Pregelatinized Breadfruit Starch (*Artocarpus altilis*) as a Novel Co-polymer for Controlled Release

**DOI:** 10.15171/apb.2019.012

**Published:** 2019-02-21

**Authors:** Adenike Okunlola, Shukuralilahi Abidemi Adewusi

**Affiliations:** Department of Pharmaceutics & Industrial Pharmacy, University of Ibadan, Ibadan, Nigeria.

**Keywords:** Chitosan, Controlled release, Microbeads, Pregelatinized breadfruit starch, Sodium alginate, Theophylline

## Abstract

***Purpose:*** The aim of this study was to prepare formulations of theophylline microbeads using pregelatinized breadfruit starch (Artocarpus altilis, family Moraceae) in combination with sodium alginate and chitosan at various polymer: drug ratios. Microbead formulations for controlled delivery of theophylline would be better alternatives to conventional dosage forms for optimized drug therapy.

***Methods:*** The native and pregelatinized starches were characterized for morphology (scanning electron microscope), crystallinity (Fourier transform intra-red spectroscopy, FTIR and X-ray diffractometer, XRD), thermal flow (differential scanning colorimeter), density and flow properties. Theophylline microbeads were prepared by ionic gelation and characterized using size, swelling index, entrapment efficiency and time required for 15% and 50% drug release (t15 and t_50_ respectively).

***Results:*** FTIR and XRD spectra revealed the orderly arrangement of granules of the semi-crystalline breadfruit starch was disrupted on gelatinization. The viscosity and flow of pregelatinized starch were enhanced. Theophylline microbeads were near spherical in shape with size range 1.09 ± 0.672 to 1.58 ± 0.54 mm. FTIR and XRD spectra confirmed there was no drug-polymer interaction. Microsphere size, swelling increased while entrapment and dissolution time (t_50_) reduced with polymer: drug ratio. The entrapment efficiency ranged from 30.99 ± 1.32 to 78.50 ± 2.37%. Optimized formulation, starch: alginate ratio 3:1 at polymer: drug ratio of 2:1, gave a prolonged dissolution time (t_50_ = 8.40 ± 1.20 h).

***Conclusion:*** Breadfruit starch was suitable as a copolymer for the controlled delivery of theophylline in microbeads which could serve as a substitute to synthetic polymers in drug delivery.

## Introduction

Microparticulate drug delivery systems are ideal for controlled delivery applications due to their biocompatibility as well as their ability to encapsulate a variety of drugs and sustain drug release.^1,2^ The selection of an appropriate coating material control the physical, chemical and release properties of the resultant microspheres.^3^ Although, several official native starches are available as coating polymers, new sources will continue to be developed as the pharmaceutical interest in starch and starch-based products grow.^2,4^ Breadfruit (*Artocarpus altilis*) is a species of flowering tree in the mulberry family (Moraceae).^5^ Despite its nutritional benefits, breadfruit remains underutilized in most tropical areas perhaps due to the perishable nature of the fruits and the limited information on commercial breadfruit orchard establishment.^6^ Currently, the use of breadfruit is attaining greater industrial importance in food applications and can be explored in the local pharmaceutical industry as a standard but cheaper alternative to imported starches.^7-9^ Among the numerous modifications that have been shown to improve the functional properties of starch, physical modification of starch granules by pregelatinization is simple, cheap and safe.^10,11^ These techniques do not require chemical or biological agents and are therefore preferred when the product is intended for human consumption, such as in drug formulations.^12,13^ Pregelatinized breadfruit starch could be used as a copolymer in the formulation of theophylline microbeads, with two established polymers, sodium alginate and chitosan. Hydrogel beads are produced by introducing drug-loaded polymeric solution into the aqueous solution of polyvalent cations to produce a three-dimensional network of ionic crossed-linked moiety.^14^ When sodium alginate and chitosan are used in the ionic gelation method, the two polymers form the polyelectrolyte complex via the ionic interaction between the carboxyl residues of alginate and the amino residues of chitosan.^15^ Thus, cross-linking of alginate and chitosan in a hydrogel can be used to provide sustained release of drugs in which the resulting systems have enhanced stability compared to those obtained with a single polymer. In a previous study, slow release formulations of imazaquin were prepared by encapsulating the herbicide in starch and chitosan beads that were reinforced with alginate.^16^ The findings showed highly porous spherical beads, with the beads containing the starch-alginate-chitosan blend being larger and less porous than those containing only the chitosan-alginate blends. In another study, microparticles of stigmasterol were obtained using a blend of polymers of sodium alginate, starch and chitosan as the coating materials through a one-stage process using the external gelation technique. Resultant microparticles were spherical, with an encapsulation efficiency of 90.42% and method yield of 94.87%.^17^ The incorporation of starch to the chitosan-alginate blends in these instances appear to modify the porosity, size and release characteristics of the formulations.

The drug of choice in this study is theophylline (1,3-dimethylxanthine),which is commonly used in the treatment of respiratory diseases such as chronic obstructive pulmonary disease (COPD) and asthma.^18^ Theophylline has a half-life of about 8 hours. The immediate release formulations of theophylline are usually administered in 2-3 doses daily. The drug is rapidly and completely absorbed after oral administration with side effects including stomach/abdominal disorder, nausea and severe vomiting. In order to optimize drug therapy, reduce the dose and frequency of drug administration, and subsequently improve patient compliance, controlled release formulations are desirable. Thus, to achieve prolonged release of theophylline, microbead formulations could be better alternatives to the conventional dosage forms. The objective of this study is to formulate theophylline microbeads of theophylline using pregelatinized breadfruit starch as co-polymer with alginate and chitosan at varying polymer: drug ratio.

## Materials and Methods

Breadfruit was purchased from a local market in Owerri, Imo State, Nigeria. Theophylline was obtained from Wuhan Hezhong Bio-Chemical Manufacture Co., Ltd., Wuhan city, China. Sodium alginate was obtained from S.D. Fine Chem (Mumbia, India). Chitosan was obtained from Sigma-Aldrich, St Louis, MO, USA. Calcium chloride was obtained from Loba Chemie Pvt. Ltd., Mumbai, India.

### Extraction and modification of breadfruit starch

Mature, unripe breadfruits were washed, peeled and cut into small pieces which were soaked in distilled water. The resulting mixture was blended to obtain a slurry that was strained through muslin cloth followed by suspending the filtrate in distilled water to enable settling. The supernatant was decanted at 12-hour intervals and the starch slurry was re-suspended in distilled water. The starch cake was collected after 72 hours and dried in a hot air oven at 50°C for 48 hours. The dried mass was pulverized and then screened through a 250-µm sieve.

To prepare the pregelatinized starch, an aqueous slurry of the dry native starch (100 g) was dispersed in 500 mL of distilled water and then heated at 100°C with constant stirring for 45 minutes. The resulting paste was dried in a hot air oven at 50°C for 48 hours and blended.

### Characterization of starches

#### Morphology

The native and modified breadfruit starches were examined for shape and size using an optical microscope (Olympus Research microscope CH20i, Olympus Optical Co, Shinjuku, Japan).

#### Fourier transform-infra red analysis

The starches were analysed by Fourier Transform-Infra Red** (**FT-IR) analysis (FT-IR Spectrum BX II by PerkinElmer, Waltham, MA, USA) in transmission mode. The transmission spectra range was 4000 - 400 cm^-1^ using 64 scans with resolution of 8 cm^-1^.

#### X-ray diffractometry

The X-ray diffraction (XRD) pattern was recorded using an X-ray diffractometer (ARL X’TRA ThermoFisher Scientific, Landsmeer, The Netherlands) with copper- cobalt radiation. The native and modified starch samples were exposed to the X-ray beam at 25°C. The scanning region of the diffraction angle (2ᶿ) was from 5° to 70^o^ at a scan rate of 12°/min. The integration time was 0.150 seconds and step was 0.03 °.

#### Differential scanning colorimetry

Thermal characteristics of the starches were studied using a differential scanning colorimeter, DSC **(**DSC 2 Mettler Toledo, Columbus OH, USA) The starch sample (about 5 mg on a dry weight basis) was heated from 60 to 300°C in sealed aluminum pans at a scanning rate of 10°C /min. Thermal transitions were characterized by onset temperature, peak temperature, conclusion temperature and the gelatinization enthalpy (ΔH).

#### Material properties of starches

#### Angle of repose

Ten grams of starch was weighed and poured through a funnel, into an opened cylinder placed on a wooden base of similar diameter. The starch was allowed to cascade into a heap. The angle of repose (*θ*) was determined as:

(1) $$\mathrm{Tan}\theta =\frac{height}{radius}$$

#### Hydration capacity

Aqueous slurry of starch samples (10% w/v) were placed in 15 mL centrifuge tubes and allowed to stand for 5 min. The starch slurries were then centrifuged at 1000 rpm for 5 min and the supernatant was discarded. The sediment was weighed to determine the gain in weight of dry sample as the amount of water sorbed by the starches.

#### pH determination

pH of a 1% w/v aqueous solution of native and modified breadfruit starches were determined (pH meter Model 720 A, Thermo Electron Corporation, Waltham, MA, USA).

#### Density

The bulk density of each excipient was determined by pouring 10 g of the powder at an angle of 45 through a funnel into a glass measuring cylinder with a volume of 50 ml. The bulk density was measured as the ratio of mass to volume occupied by powder. The tapped density was measured by applying 100 taps to 10 g of powder in a graduated cylinder. The solvent pycnometer method was used to determine the density of the starches using xylene as the non-solvent.^19^

#### Viscosity

The viscosity of a 1 %w/v aqueous slurry of each starch was determined using Brookfield rheometer (DV-III + model, Brookfield Engineering, Middleborough, MA, USA) using CPE 40 and spindle sizes of 2 and 4 at 27 ± 2°C.

All determinations were done in triplicates with the values of mean and standard deviation presented.

#### Formulation of starch-alginate-chitosan-based microbeads of theophylline using pregelatinized breadfruit starch as a copolymer

#### Preformulation studies

Microbead formulation trials were carried out by varying the ratio of the pregelatinized breadfruit starch to sodium alginate, concentration of calcium chloride solution, amount of chitosan, ratio of total polymer to drug, stirring speeds and curing times. Optimized formulations obtained were prepared as described below:

#### Preparation of theophylline microbeads

A 2% w/w aqueous solution of sodium alginate and pregelatinized starch blend was prepared (starch: alginate ratio of 1:1). To the alginate-starch polymer blend was added 1 g of theophylline (to obtain a total polymer: drug ratio of 2:1). Stirring was done continuously for 20 min using a magnetic stirrer. The drug-loaded polymer solution was manually extruded (using a syringe with needle size 21G) into 200 ml of calcium chloride (10 %w/w) that contained 0.2% w/v chitosan solution ( in 1%v/v dilute acetic acid) inside a 250-mL beaker. Stirring was continuous for 15 minutes at room temperature (25 ± 2°C); the beads formed were collected, washed with distilled water to eliminate the excess calcium ions and then dried overnight at 50°C. The composition of the prepared formulations was varied changing the ratio of starch: alginate (2:1) and polymer: drug (1:1) while formulations containing alginate alone were used as standard.

#### Characterization of starch-alginate-chitosan microbeads of theophylline

#### Microencapsulation yield

The microencapsulation yield was determined by the ratio of the dry weight of the microbeads that were obtained to the total weight of solid raw materials (drug and polymers) used in the preparation of microbeads.

#### Morphology

The morphology and surface characteristics of the microbeads were observed using a scanning electron microscope, SEM (Hitachi Model S-2460N Taichung, Taiwan) at an accelerating voltage of 25 kV.

#### X-ray diffractometry

The XRD pattern was recorded using an X-ray diffractometer (ARL X’TRA ThermoFisher Scientific, Landsmeer, The Netherlands) with copper- cobalt radiation. The scanning region of the diffraction angle (2ᶿ) was from 5^o^ to 70^o^ at a scan rate of 12 ᵒ/min.

#### FT-IR analysis

The drug, pregelatinized starch, alginate, chitosan and drug-loaded starch-alginate-chitosan microbeads were analysed by FT-IR (FT-IR Spectrum BX II by PerkinElmer, Waltham, MA, USA**)** in transmission mode. Pellets of the samples were prepared by finely grounding with KBr under a hydraulic pressure of 600 dynes/m^2^. The spectra were scanned between 4000 to 400 cm^-1^ (32 scans with resolution of 8 cm^-1^).

#### Swelling

Accurately weighed amounts of beads were placed in glass vials containing 5 mL of distilled water. After two hours, the samples were recovered, gently wiped with absorbent paper and weighed again. The percent swelling was determined using:

(2) $$Sweling\%=\frac{W_{t}-W_{0}}{W_{0}}\times 100$$

Where *w*_t_ refers to the weight after 2 hours in water and *w*_0_ refers to the initial weight of the microbeads.

#### Drug loading

Drug loading was calculated using:

(3) $$Drug\;loading(\%)=\frac{Weight\;of\;drug\;in\;microspheres}{Weight\;of\;drug\;in\;microspheres}\times 100$$

#### Entrapment efficiency

Drug-loaded microbeads (50 mg) were accurately weighed, crushed and suspended in 100 mL of phosphate buffer pH 6.8 (prepared by dissolving 68.04 g of potassium dihydrogen phosphate, KH2PO4 and 8.96 g of sodium hydroxide into 10 L of distilled water. After 24 hours, the solution was filtered and the filtrate was appropriately diluted with the buffer and analyzed using UV/VIS spectrophotometer (Spectrum lab 752s UV-VIS spectrophotometer, Changsha Hunan, China) at 270 nm. The drug entrapment efficiency (E) was determined as follows:

(4) $$\text{E(\%)}=\frac{\text{Actual drug content}}{\text{Theoretical drug content}}\times 100$$

#### Drug content

One hundred milligrams of microspheres were measured into a 100-mL volumetric flask. Water was added to the 100-mL mark and the microspheres were suspended in phosphate buffer pH 6.8, shaken vigorously and then left for 24 hours at room temperature while shaking intermittently. The supernatant obtained was centrifuged and the drug content was determined by UV spectroscopy using a UV spectrophotometer (Spectrum lab 752s UV-VIS spectrophotometer, Changsha Hunan, China) at 270 nm.

#### Drug release study

Microbeads containing 50 mg of theophylline were accurately weighed and suspended into 900 mL of phosphate buffer pH 6.8. The *in vitro* dissolution studies were carried out using the paddle method (USP XXXVI), rotated at 50 rpm in 900 mL of phosphate buffer pH 6.8 and maintained at 37 ± 0.5°C. Ten milliliters of dissolution medium were withdrawn at time intervals and replaced with equal amounts of fresh medium. The amount of theophylline released at each time interval was determined at a wavelength of 270 nm, using a UV spectrophotometer (Spectrum lab 752s UV-VIS spectrophotometer, Changsha Hunan, China). Determinations were made in triplicate. The results of the drug release for the formulations was fitted to zero order, first order (ln *Qt* = ln *Q*_0_ + *K*_1_*t*), Higuchi (*Q* = K_H_√t), Hixon-Crowell (*Q*_0_^1/3^– *Q*_t_^1/3^= *K t*) and Korsemeyer–Peppas (*Q*_t_ /*Q*_∞_ = *K t*^n^) kinetic equations. The model of best fit was identified by comparing the values of the correlation coefficients.

#### Statistical analysis

A statistical analysis was conducted to compare the effects of the polymers on the microbead properties using the analysis of variance (ANOVA). At a 95% confidence interval, probability, p values less than or equal to 0.05 were considered significant.

#### Theory

In this study, the matrix of starch-alginate polymer blend was modified with chitosan. The mechanism of interaction between starch and alginate blend was intermolecular hydrogen bonding. The incorporation of starch provides a filling material that reduces the permeability of the otherwise porous alginate microbeads while aiding stability. The complexation that occurs between alginate and chitosan is via an ionic interaction between the carboxyl residues of the alginate and the amino terminals of chitosan.^20^ In addition, this interaction produces a reduction in the porosity of the alginate beads thereby minimizing the leakage of the encapsulated drug. Further interaction occurs via intermolecular hydrogen bonding, similar to the mechanism of starch—alginate cross linking. The chitosan acts a coat that enhances the rigidity of the microbeads while reducing their permeability.

## Results and Discussion

### Characterization of native and pregelatinized breadfruit starches

**Figure 1:**
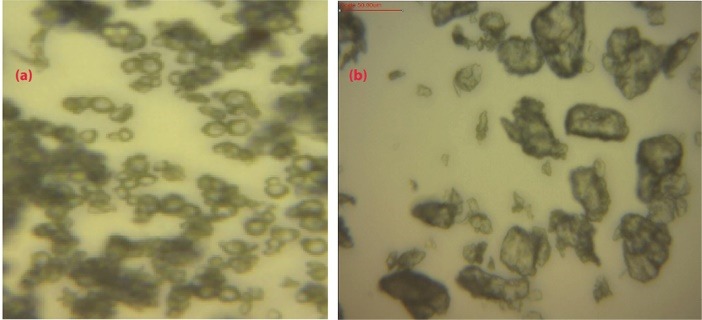
Photomicrographs of (a) native and (b) pregelatinized breadfruit starches (x100).

**Figure 2:**
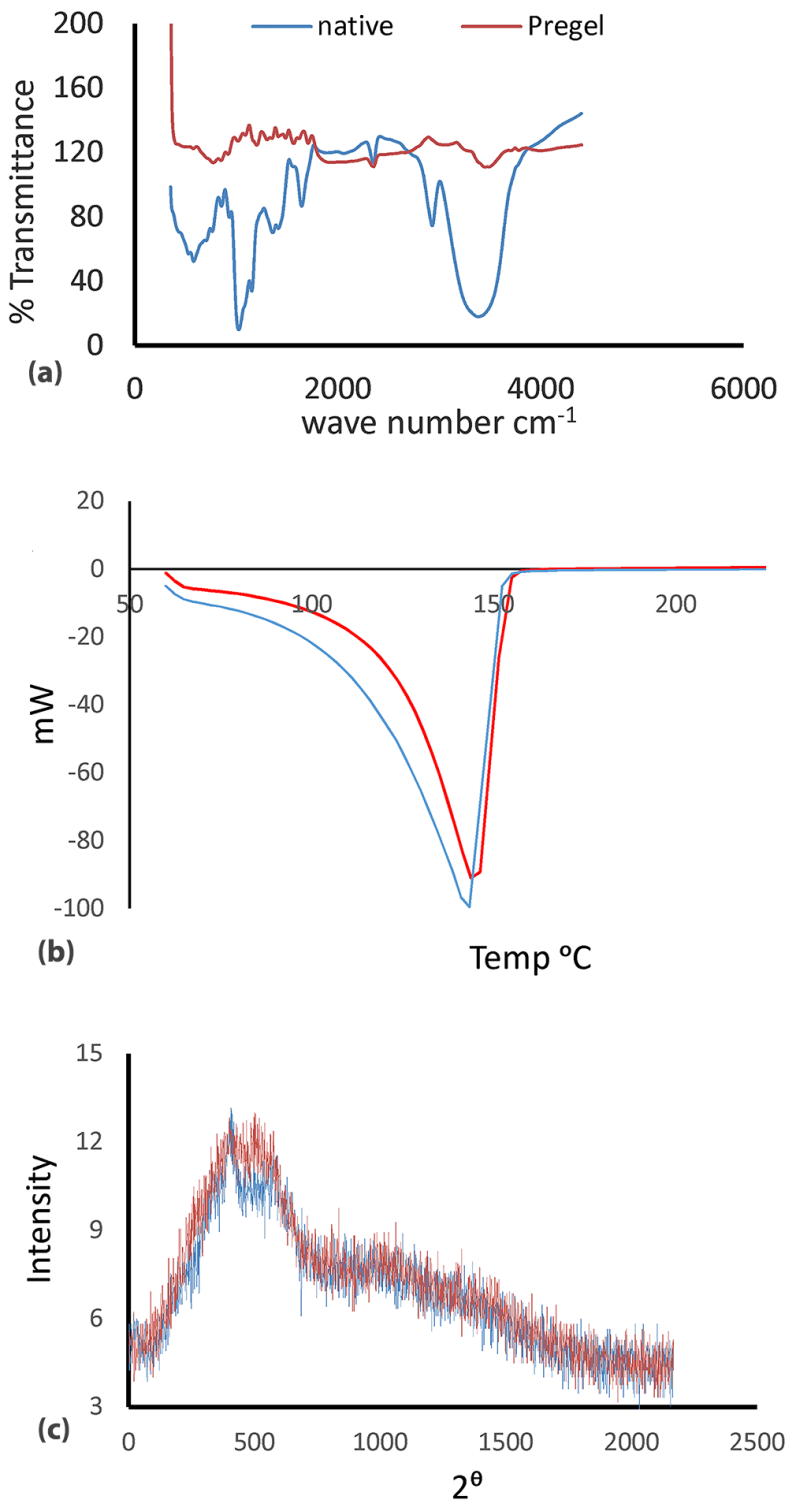
(a) Fourier transform infra-red (FTIR) spectra, (b) differential scanning colorimetry (DSC) endotherms and (c) X-ray diffraction (XRD) spectra of native and pregelatinized breadfruit starches.

The yield of starch obtained from breadfruits was 38.23%w/w. This appears to be a reasonably good yield considering the high water content of the fruit.^9,21^ Photomicrographs of native and pregelatinized breadfruit starch are shown in Figure 1. The starch granules of the native breadfruit starch were spherical in shape with granule size of 8.44 ± 4.00 µm. When modified by pregelatinization, the starch granules became larger and irregular in shape. The FTIR and XRD spectra of the native and pregelatinized breadfruit starch are shown in Figures 2a and  2b respectively, while DSC endotherms of native and pregelatinized starches are shown in Figure 2c. FTIR spectra revealed that the orderly arrangement of granule structure of the native starch was disrupted when heated in water resulting in gelatinization. This is reflected by the reduction in the band intensity at 1042 cm^-1^ and is correlated with the XRD results which revealed disruption of the internal order of the starch granules in the pregelatinized form. DSC results showed endothermic peaks with the onset, peak, conclusion temperatures and enthalpy for the native breadfruit starch at 59.86°C, 142.81°C, 150.67°C and 1332.18 J/g respectively, whereas those for the pregelatinized starch were 61.60°C, 144.77°C, 152.58 °C and 1686.12 J/g respectively.

The material and physicochemical properties of native and breadfruit starches are presented in Table 1. The hydration capacity of the starches at room temperature (27 ± 2°C) showed higher values for the pregelatinized breadfruit starch than for the native starch owing to the loose structure of the modified starch in comparison to the firm structure of the native starch. An amylose/amylopectin ratio of 27.68: 72.32 has been reported for breadfruit.^22^ During pregelatinization of starch granules, the amylose is precipitated and releases amylopectin which is responsible for swelling and water retention ability. When in contact with water, pregelatinized starch readily forms a gel and the strength of the gel is related to the amylose component. On the other hand, the cohesiveness of the gel depends on the amylopectin component.

**Table 1:** Material and physicochemical properties of native and pregelatinized breadfruit starches

**Starch**	**Particle size** **(µm)**	**Angle of repose (ᵒ)**	**Hydration capacity (%)**	**pH**	**Particle density** **(gcm** ^-3^	**Bulk density** **(gcm** ^-3^	**Tapped density** **(gcm** ^-3^	**Carr’s index** **(%)**	**Hausner’s** **ratio**
Native	8 .44± 4.00	66.92 ± 1.31	190.50±5.50	4.73 ± 0.18	1.46 ± 0.02	0.32 ± 0.01	0.54 ± 0.02	41.25 ± 0.93	1.7 ± 0.03
Pregel	34.45 ± 6.70	58.67 ± 0.63	281.10±4.40	3.93 ± 0.01	1.49 ± 0.02	0.65 ± 0.03	0.95 ± 0.06	32.07 ± 7.69	1.48 ± 0.17

Data are shown as mean ± SD (n = 3).

The particle, bulk and tapped densities of the modified starch were significantly higher than those of the native starch (*P* < 0.05). The Carr’s index and Hausner’s ratio values were obtained from the bulk and tapped densities. The values revealed that modification of native breadfruit starch by pregelatinization improved the flowability of the powder but reduced its compressibility. This enhancement of flow in the pregelatinized starch was further confirmed using the angle of repose. The higher the angle of repose, the more cohesive the powder and the poorer the flow of the powder.

The values of viscosity of the native and pregelatinized breadfruit starches are presented in Table 2. The viscosity values give a measure of a material’s resistance to gradual deformation by shear stress or tensile stress. The viscosity of the pregelatinized starch was higher than that of the native breadfruit starch at 50 rpm. However, It was observed that as the speed increased to 100 rpm, the viscosity of the modified starch decreased with a spindle size 2 but remained the same with spindle size 4.

**Table 2:** Viscosity of native and pregelatinized breadfruit starches at 50 and 100 rpm

**Starch**	**Spindle size**	**Speed (rpm)**	**Torque (%)**	**Viscosity** **(cps)**
Native	2	50	0.90	7.20
		100	2.25	9.00
	4	50	0.10	4.00
		100	0.30	6.00
Pregelatinized	2	50	0.85	7.60
		100	2.15	8.60
	4	50	0.10	4.00
		100	0.30	6.00

### Characterization of starch-alginate-chitosan microbeads of theophylline

**Figure 3:**
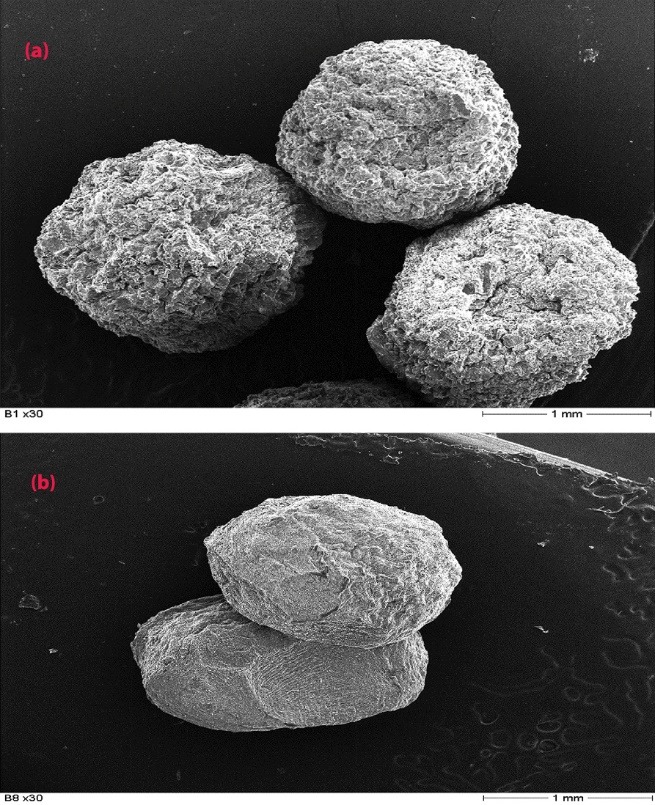
Scanning electron micrographs (SEM) of: (a) starch-alginatechitosan microbeads of theophylline containing pregelatinized breadfruit starch and (b) alginate-chitosan microbeads of theophylline (x30).

The ionic gelation method is a simple, reproducible process devoid of the use of organic solvents and high temperatures. The yield of microbeads was 91.25% w/w. Scanning electron images (SEM) for the microbeads are shown in Figure 3. The SEM images revealed that the starch-alginate-chitosan theophylline microbeads (B_1_-B_6_) were near spherical in shape. Their size ranged from 1.09 ± 0.672 to 1.58 ± 0.54 mm as presented in Table 3. The ranking of microbead size for all formulations was in the order of B_8_ < B_7_ < B_2_ < B_1_ < B_4_ < B_3_ < B_6_ < B_5._ The microbeads containing breadfruit starch were larger than those containing alginate only (B_7_ and B_8_). The size appeared to increase with starch content. This could be due to the increased viscosity of the starch-alginate polymer blend with increased amounts of incorporated starch, leading to an increase in the amount of polymer-drug droplets extruded into the chelating medium, thus forming larger microbeads.

**Table 3:** Formulations and properties of theophylline microbeads

**Batch**	**Starch:** **Alginate**	**Polymer:** **drug**	**Yield** **%**	**Size** **mm**	**Swelling** ** index**	**Drug content %**	**Drug** **loading %**	**Entrapment** ** %**	**t**_15_ **h**	**t**_50_ **h**
B1	1:1	2:1	85.4 ± 3.45	1.15 ± 0.67	1.37 ± 0.09	16.67± 2.85	19.52 ± 1.60	49.38 ± 2.55	0.75 ± 0.04	7.40 ± 0.40
B2	1:1	1:1	80.10 ± 2.65	1.09 ± 0.72	1.28 ± 0.01	22.00± 1.78	27.50 ± 0.85	44.90 ± 6.20	1.50 ± 0.11	4.80 ± 0.45
B3	2:1	2:1	88.80 ± 5.67	1.35 ± 0.83	1.49 ± 0.12	21.76± 2.05	26.25 ± 2.77	65.29 ± 5.32	1.60 ± 0.14	7.80 ± 0.77
B4	2:1	1:1	83.30 ± 3.73	1.25 ± 0.76	1.39 ± 0.12	25.99± 2.12	31.20 ± 3.10	51.97 ± 4.03	0.60 ± 0.03	5.60 ± 1.44
B5	3:1	2:1	92.00 ± 4.60	1.58 ± 0.44	1.86 ± 0.12	26.17± 1.55	28.45 ± 1.55	78.50 ± 2.37	1.80 ± 0.07	8.40 ± 1.20
B6	3:1	1:1	90.44 ± 2.50	1.42 ± 0.54	1.61 ± 0.12	34.50± 2.95	38.01 ± 3.15	69.00 ± 1.60	0.75 ± 0.02	6.60 ± 0.54
B7	0:1	2:1	89.75± 3.25	1.04 ± 0.76	1.26 ± 0.18	12.29± 1.28	13.69 ± 0.27	40.97 ± 0.33	1.00 ± 0.06	5.00 ± 1.76
B8	0:1	1:1	92.33 ± 3.76	0.98 ± 0.55	1.01 ± 0.12	15.49± 0.95	16.78 ± 0.15	30.99 ± 1.32	0.85 ± 0.02	4.00 ± 0.39

Note: t_15_ is the time taken for 15% of drug to be released; t_50_ is the time taken for 50% of drug to be released.

**Figure 4:**
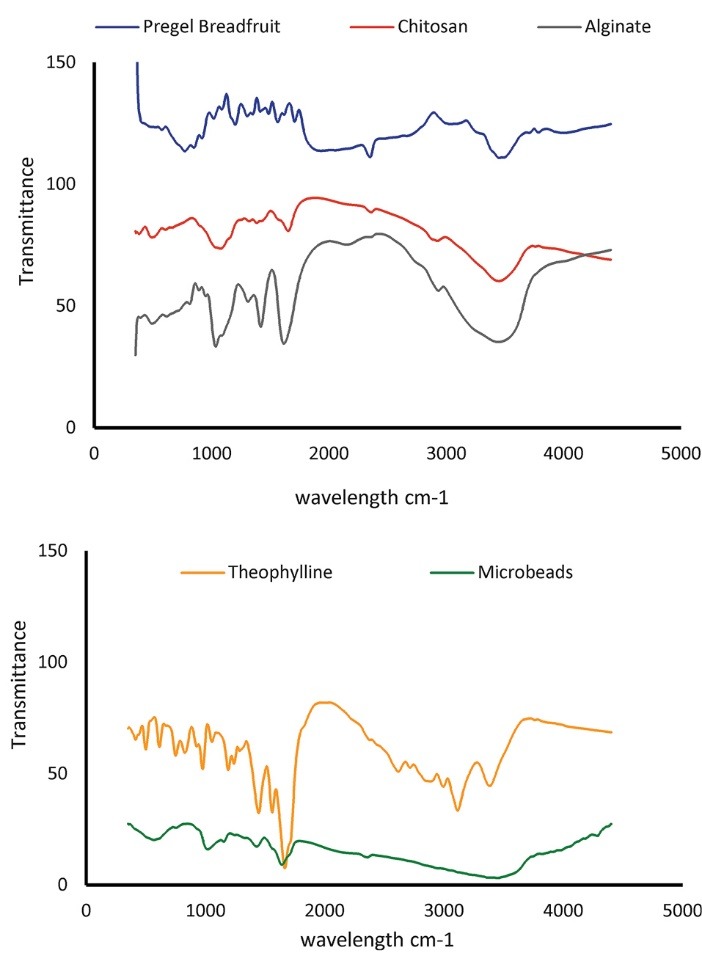
Fourier Transform Infra-Red (FTIR) spectra of pregelatinized breadfruit starch, alginate, chitosan, theophylline and starch-alginatechitosan- based microbeads of theophylline.

The FTIR spectra of sodium alginate, chitosan, pregelatinized breadfruit starch, theophylline and the starch/alginate-based theophylline microbeads are shown in Figure 4. The FTIR spectra showed that there was no disruption or interaction between theophylline and the polymers used in the microsphere formulation. It also showed that theophylline was well entrapped within the co-polymer blend. The broad absorption band observed between 3600 and 3200 cm^−1^ is due to the hydroxyl group O−H stretching vibrations which is characteristic of all the natural polysaccharides. The strong asymmetric stretching absorption band at 1650 cm−1 and the weaker symmetric stretching band near 1430 cm^−1^ are due to the presence of carboxylate anions. The band at 1993 cm^−1^ is also assigned to asymmetric stretching of −COO− groups. The peak at 1058 cm^−1^ was shifted to lower wave-numbers in the microbead formulation. The bands observed at 1092 and 1043 c m−1 are characteristic of the C−O−C link. For the FTIR spectrum of chitosan, two weak peaks were observed between 2400 and 2350 cm^−1^ that were shifted to 2900 cm^−1^ in the microbead formulation. The characteristic medium intensity band at 4000 - 3500 cm^−1^ were also observed for chitosan. The FTIR spectrum of theophylline showed a broad and weak transmission band of N-H stretching vibration at 3000 cm-1. The N-H bending vibration was observed at 1663 cm^-1^ while stretching vibrations were observed at 1561 cm^-1^. The carbonyl group (C=O) was observed at 1705 cm^-1^ whereas the C-H stretching vibrations superimposed upon the N-H broad bend at 3054 and 2983 cm^-1^.

**Figure 5:**
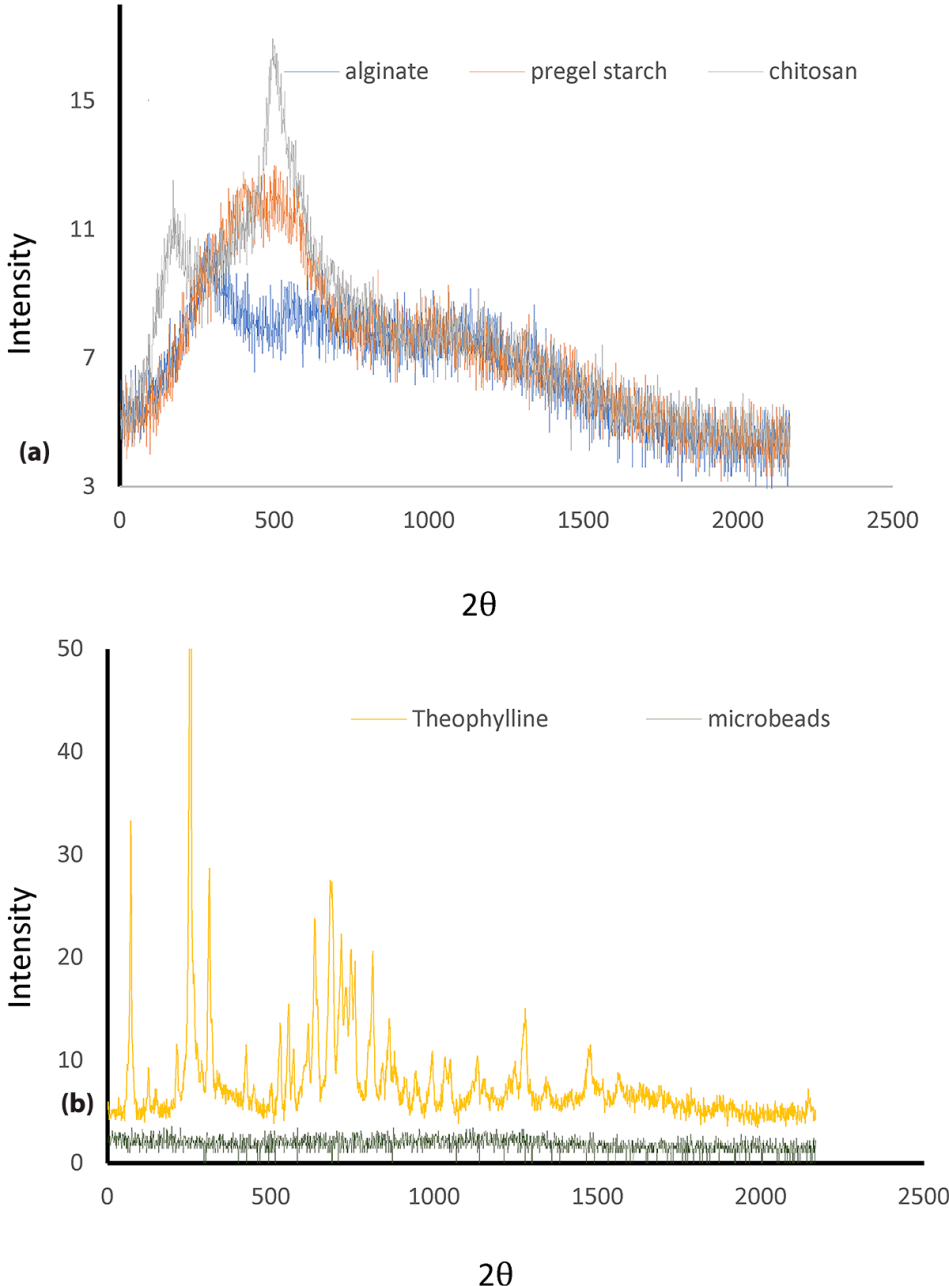
X-ray Diffraction (XRD) spectra of pregelatinized breadfruit starch, alginate, chitosan, theophylline and starch-alginate-chitosan -based microbeads of theophylline

As revealed in the XRD pattern of theophylline in Figure 5, the diffraction peaks of the drug showed its crystalline form. The XRD pattern of the microbeads did not show any peak like those observed for the amorphous starch, alginate and chitosan, confirming that blend achieved good drug entrapment. XRD patterns showed that theophylline did not significantly alter the amorphous polymeric network, implying that breadfruit starch-alginate-chitosan microbeads can be used as a suitable controlled-release carrier for theophylline.

The values of the swelling and entrapment efficiency of theophylline microbeads are also presented in Table 3. The swelling index values increased with starch content and polymer to drug ratio. The ranking of swelling was as follows: B_8_ < B_7_ < B_2_ < B_1_ < B_4_ < B_3_ < B_6_ < B_5._ Microbeads containing a starch to alginate ratio of 3:1 (Batches B_5_ and B_6_ ) had the highest swelling and this can be attributed to their high starch content. The entrapment efficiency values were within the range of 44.90 ± 6.20 to 78.50 ± 2.37%. The amount of drug entrapped increased with starch content and entrapment efficiency was in the same rank order as swelling. These results indicate that the presence of starch enhanced entrapment when compared to microbeads containing sodium alginate alone.

**Figure 6:**
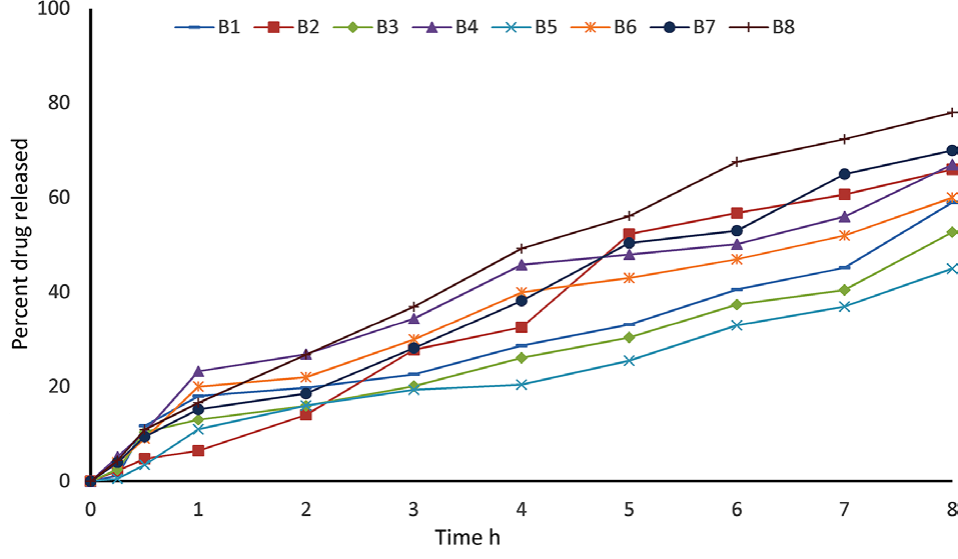
Dissolution plots of microbead formulations of theophylline containing pregelatinized breadfruit starch, sodium alginate and chitosan.

The dissolution profiles of the microbeads are presented in Figure 6. From the plots, the values of t_15_ and t_50_ (time required for 15 and 50% drug release respectively) were determined and are also presented in Table 3. The release behavior of microspheres has been shown to be dependent on parameters such as the polymer type, particle size and drug-matrix interactions within the microsphere system.^23-27^ The release pattern of the theophylline microbead formulations showed that Batches B_7_ and B_8_ (sodium alginate alone) as well as B_1_, B_4_ and B_6_ had an initial burst release as observed from the short period of t_15_ (<60 minutes).^23,28,29^ This was followed by slow release of the drug. Microbeads with smaller sizes released a higher percentage of the drug which may be due to their larger surface areas. The presence of starch contributed to the delayed release of theophylline due to its matrix structure which reduced porosity and decreased the leakage of the encapsulated drug. Chitosan is also known to form a cross-link with sodium alginate, leading to blockage of pores on the surface of the microbeads, thereby sustaining drug release. Chitosan also enhanced the mechanical strength of microbeads.^16,30^ The ranking of the batches in terms of t_50_ was: B_ 5_ > B_3_ > B_1_ > B_6_ > B_4_> B_2_> B_7_ > B_8_. The percentage of drug released decreased with increasing amount of starch in formulation and with increase in polymer: drug ratio. The optimum formulation was B_5_ containing starch: alginate ratio 3:1 and polymer: drug ratio of 2:1 which gave a controlled dissolution time t_50_ of 8.40 ± 1.20 hours and entrapment efficiency of 78.50 ± 2.37 % which were significantly (*P* < 0.05) higher than those of the formulations containing alginate alone.

Release of a solid drug from the microsphere matrix involves the simultaneous penetration of the surrounding liquid, the dissolution of the drug, and the leaching out of the drug through interstitial channels or pores. The kinetics of drug release is important due to their influence on drug bioavailability. The physicochemical properties of the drug and the polymer have been shown to govern the release of drug from formulations which could affect their release kinetics.^31^ The drug release kinetics of the batches were simulated using: zero order, first order, Higuchi,^32^ Hixson-Crowell,^33^ Korsmeyer-Pepppas (Table 4).^34^ Drug release from formulations B_1_, B_3_ and B_7_ generally fitted the zero order implying that the process is constant and independent of the initial concentration of the drug in the delivery system. Only Formulation B_2_ fitted the Korsmeyer-Peppas model. The Korsmeyer-Peppas model is a simple relationship that describes drug release from a polymeric system in which the value of n characterizes the release mechanism of the drug. When 0.45 ≤ n, this corresponds to a Fickian mechanism; when 0.45 < n < 0.89, this corresponds to non Fickian transport. When n = 0.89, this corresponds to a case II (relaxational transport) and n >0.89 to a super case II transport.^34^ From the values of the slopes, the drug release mechanism from Formulation B_2_ is considered to be super case II transport. On the other hand, Formulations B_4_ and B_5_ fitted the Hixson-Crowell kinetic model, which describes the release from a system which there is a change in the surface area and the diameter of particles.^33^ Formulations B_6_ and B_8_ fitted the first order kinetic model, implying that the release of theophylline was from a porous matrix.^35^

**Table 4:** Correlation coefficients obtained using different kinetic models (n = 3)

**Batch**	**Zero order**	**First order**	**Higuchi**	**Hixson-Crowell**	**Korsmeyer**	
	**R** ^ 2 ^	**R** ^ 2 ^	**R** ^ 2 ^	**R** ^ 2 ^	**R** ^ 2 ^	**n**
B_1_	0.9519*	0.9341	0.9285	0.9068	0.8232	0.8556
B_2_	0.9801	0.9774	0.9237	0.9535	0.9861*	1.0213
B_3_	0.9728*	0.9574	0.9369	0.9395	0.9237	0.7193
B_4_	0.9439	0.9682	0.9801	0.9830*	0.9675	0.6785
B_5_	0.9671	0.9707	0.9430	0.9708*	0.9087	1.0753
B_6_	0.9600	0.9834*	0.9812	0.9833	0.9677	0.7164
B_7_	0.9904*	0.9783	0.9492	0.9633	0.9827	0.7815
B_8_	0.9847	0.9921*	0.9745	0.9886	0.9912	0.7958

*Highest correlation coefficient for batch.

## Conclusion

The ionic gelation method was found to be efficient and reproducible for the formulation of theophylline microbeads when pregelatinized breadfruit starch was used as a co-polymer in different blend combinations with sodium alginate and chitosan and at different polymer to drug ratios. The drug release profile of the microbeads showed dependence on the amount of starch used and the polymer drug ratio. Further research may still be required using *in vivo* studies to confirm the versatility of pregelatinized starch of breadfruit as a sustained release polymer in the preparation of microbead formulations of theophylline and other drugs. Future research could also focus on formulating and evaluating starch-based theophylline microbeads in the form of dry powders to produce aerosols intended for inhalation.

## Ethical Issues

Not applicable.

## Conflict of Interest

The authors declare that there is no conflict of interest.
